# Correction: Batch-produced, GIS-informed range maps for birds based on provenanced, crowd-sourced data inform conservation assessments

**DOI:** 10.1371/journal.pone.0295634

**Published:** 2023-12-05

**Authors:** Ryan M. Huang, Wilderson Medina, Thomas M. Brooks, Stuart H. M. Butchart, John W. Fitzpatrick, Claudia Hermes, Clinton N. Jenkins, Alison Johnston, Daniel J. Lebbin, Binbin V. Li, Natalia Ocampo-Peñuela, Mike Parr, Hannah Wheatley, David A. Wiedenfeld, Christopher Wood, Stuart L. Pimm

Shortly before this *PLOS ONE* article [1] was accepted, a similar article was published by Palacio et al. in *Diversity and Distributions* [2]; a preprint of the Palacio et al. article was posted on bioRxiv in May 2020 [3]. The authors did not cite [3] in [1].

Both articles [1, 2] address similar research questions and use occurrence data, habitat layers, and interpolation methods to build species distribution maps, but they used distinct methods (nearest neighbor interpolation, alpha hulls, and gridded absences in [1] and inverse distance weighting, minimum convex polygons, and point absences in [2]). [2] used occurrence data from an online data repository to create new maps, and [1] combined crowd-sourced presence points with published range maps [4, 5]. Additionally, the *PLOS ONE* study included more details and discussion of limitations of its reported model as compared to the *Diversity and Distributions* study, and found that whether species’ area of habitat is larger or smaller than the original range map differs across IUCN Red List categories.
