# Supplementary material for: Batch-produced, GIS-informed range maps for birds based on provenanced, crowd-sourced data inform conservation assessments
Source: PLoS One. 2021 Nov 24;16(11):e0259299. doi: 10.1371/journal.pone.0259299 (PMC8612558; doi:10.1371/journal.pone.0259299)
Supplement: S1 Text — (DOCX) [file pone.0259299.s001.docx]

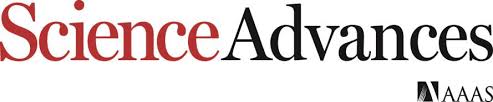


advances.sciencemag.org/cgi/content/full/sciadv.[ms.no.]/DC1

Supplementary Materials for

**Batch-produced, GIS-informed range maps for birds based on provenanced, crowd-sourced data**

Ryan M. Huang, Wilderson Medina, Thomas M. Brooks, Stuart H. M. Butchart, John Fitzpatrick, Claudia Hermes, Clinton N. Jenkins, Alison Johnston, Daniel J. Lebbin, Binbin, V. Li , Natalia Ocampo-Peñuela, Mike Parr, Hannah Wheatley, David Wiedenfeld, Christopher Wood, Stuart L. Pimm

*Corresponding author. Email: [stuartpimm@me.com](mailto:stuartpimm@me.com)

**This PDF file includes:**

Supplementary Text

Figs. S1 to S3

References (22 to 23)

**Other Supplementary Materials for this manuscript include the following:**

Table S1-S2

Code S1

Supplementary Text

Combining expert system data with crowd-sourced data

Central to this paper’s effort is combining already published data with data we extract from eBird. Three such actions are involved: geographical ranges, habitat choices, and elevations. We consider each in turn. We conclude with a discussion of why we did not include GBIF data, including historical records in this paper. (We explore what these data tell us in work in progress.)

Geographical ranges

Our methods start with the union of the previously published maps and the alpha hull around the eBird observations. The previously published maps are not transparent with respect to their sources. So, particularly when those maps show areas outside of the limits of eBird data, they raise the obvious question about their provenance. For areas and species that we know best, it was sometimes a simple matter of searching the literature for the name of those areas. For instance, Desengano State Park is an extensive area of forest within the Mata Atlântica of Brazil that has been completely ignored by eBirders. There are literature reports from ornithological expeditions there, however (22). Searching all such records across Central and South America would have been impossible and, in any case, they are not geo-referenced. Taking the union of maps and hulls was the only practical solution.

Elevations

Elevational data come from Birdlife and Stotz et al. (23). They are usually identical, suggesting the readily available data from the latter is the source. Again, we chose to use the union of published estimates and eBird data, taking the minimum for the lower elevation and the maximum for the higher.

Extracting eBird elevations raise two issues. eBird data provide latitude and longitudes. First, assume that these are an accurate estimate of where the observer saw the species. Our methods extract the elevation of each point from a digital elevation map (DEM). The finest resolution widely available is a 90m by 90m pixel. There is also a 1km resolution product, averaging the elevations across its extent.

These two sets of estimates do not always agree (Fig. S1). Moreover, the discrepancies differ for maximum and minimum elevations. The greatest differences for the former come from lowland species that penetrate into the Andes along deep, narrow valleys. The 1km average elevation might include the mountains nearby. The reverse applies for minimum elevations.

We chose to use the elevations estimated from the 1km resolution DEM. eBird locations are not spot counts, but often involve explorations along a few kilometers of road or pathways. (We only exclude observations that cover more than 7km.) Moreover, that hypothetical observation in valley bottom might be from there but could be of bird seem several hundred meters away up the valley side. It might be flying overhead. Furthermore, there might be errors in where the observer recorded the observation.

Second, we can ask how the eBird estimates compare to those previous published. About one third of the estimated upper limits fall below those previously published. Birdwatchers look for the species where they are likely to find them, not to expand their upper limits. For these, we use the published upper limits. But, as Fig. S2 shows, our upper estimates are sometimes considerably higher and our lower estimates considerably lower.

How credible are these extreme values? One of the most extreme examples, the black-capped tinamou, *Crypturellus atrocapillus*, was pointed out by author JF, who is very familiar with the species. The previously published upper limit is 900m. Our estimate on a 1km DEM was 3680m. This species occurs in deep valleys well into the Andes. The estimate of its upper elevation on the 90m DEM was 2748m. Using that DEM, of the 178 records of this species, there are three records above 2300m and 30 more between 900m and 2300 m. For example, there is one record from an official Peruvian bird guide, with over 1,000 species reported for Peru on eBird, where the location is described as “2200 to 2500m” above sea-level.

Another example, is the purple-chested hummingbird, *Amazilia rosenbergi*, has a published upper elevation of 200m, compared to our estimate of 2258 using the 90m DEM. All but 99 of the138 eBird records are above 200m, 23 are above 1000m.

In short, some of the extreme elevation records seem quite plausible.

Table S2, lists the upper and lower elevations we estimate from both 1km and 90m mapping, and provides the complete distribution of observations from the latter by 100m bands. It also includes estimates from BirdLife and Stotz.

The question remains is how much difference does it make to our estimates of Areas of Habitat when we use these values as compared to the much smaller published upper limits?

Table S3 examines eight species that have the largest discrepancies between our range estimates and the published ones. The increase in Area of Habitat using our wider ranges over the published ones ranges from 9% to 117%. The lowest value is the line-cheeked spinetail, *Cranioleuca antisiensis,* which changes its upper elevation with latitude. We call this the “seedsnipe” problem — species that are near sea-level in Patagonia, but on mountain tops near the equator. Largely, this isn’t a problem for the species we consider here, because we select only those species with relatively small ranges.

The differences for *Crypturellus atrocapillus* — 32% — and *Amazilia rosenbergi* — 62% — must be viewed in the light that their published ranges are exceeded by many observations. Fig. S3 shows how the maps appear based on the two sets of range estimates.

Habitat choices

Mapping habitat is fraught with assumptions. As discussed in the main text, some species will have specific requirements — not just forest, for example, but special forests, such as those with bamboo or along rivers. Our estimates of Area of Habitat will surely be overestimates for many species.

BirdLife lists habitats for the species. For about 90% of the species, “forests” of one kind of another are at least one of the major habitat preferences. Our methods allow estimation of how much forest cover each species selects. These estimates vary as expected, being higher for species of moist forests and lower for species of dry forest. We excluded values of zero forest cover because there were often such observations for well-known forest-dependent species. Likely, the observer was on a road, perhaps in a clearing, or might have been unable to get a GPS reading where (s)he was. The problem with this is that some species select grasslands, deserts, or upper elevation shrublands.

For these we do not have confidence in our estimates of Area of Habitat using forest cover. We can still estimate how much of the species range occurs within the elevational limits of the species, of course.

Table S1 lists all the habitats within the Birdlife data base. We classified 96 species as being open area species, based on their major habitats being grassland, shrubland — and especially these habitats at high elevations, plus a few other specialized habitats such as peatlands, mountain peaks, and the intertidal. For the great majority, we estimated the forest cover at below 10%, but for a few it was higher, because our methods excluded zero forest cover estimates.

These open area species require approaches to identifying their habitat that are beyond this paper. Some 18 of the 96 are threatened — essentially the same percentage as for the forest species.

Data exclusions: GBIF and museum specimens

The maps we produce show GBIF data, some of which are from museum specimens, as well as more recent records. The also show eBird records that are classified as “historical.” In all these cases, we do not use these records for defining the alpha hulls or estimating forest cover and elevational limits. The principal reason for their exclusion is that they involve different protocols and we would have to create a different set of criteria for including or excluding them. In most cases, these records fall within the polygons we create. Sometimes, they appear to be responsible for outlying polygons for a species distribution that the published maps produce.

For yet others, the records suggest to us locations where the species may no longer survive because we do not see suitable habitat. This suggests that looking at historical records may provide insights into how habitat is shrinking and thus how species are likely declining. That must wait for another paper, since it involves substantially more analyses.


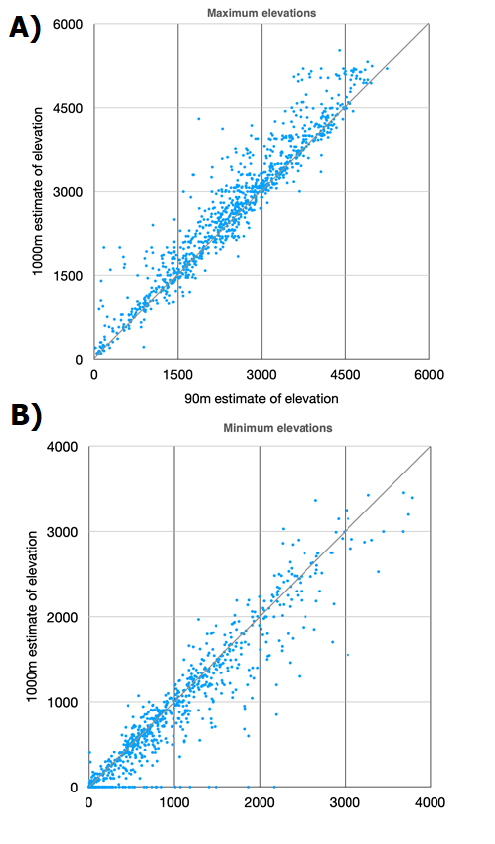


Fig. S1. A) Maximum and B) minimum elevations of species using locations on a 1km resolution DEM versus a 90m resolution DEM. (Some of the zeros on the bottom graph are likely missing values.)


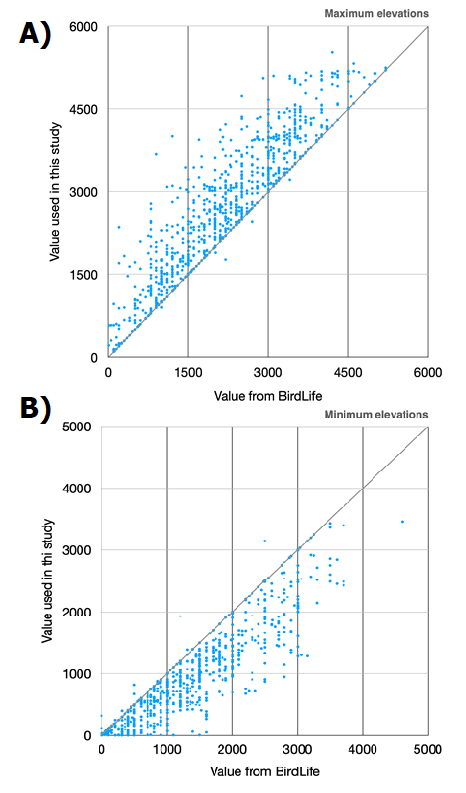


Fig. S2. A) Maximum and B) minimum elevations of species using locations on a 1km resolution the published limits from BirdLife.

*
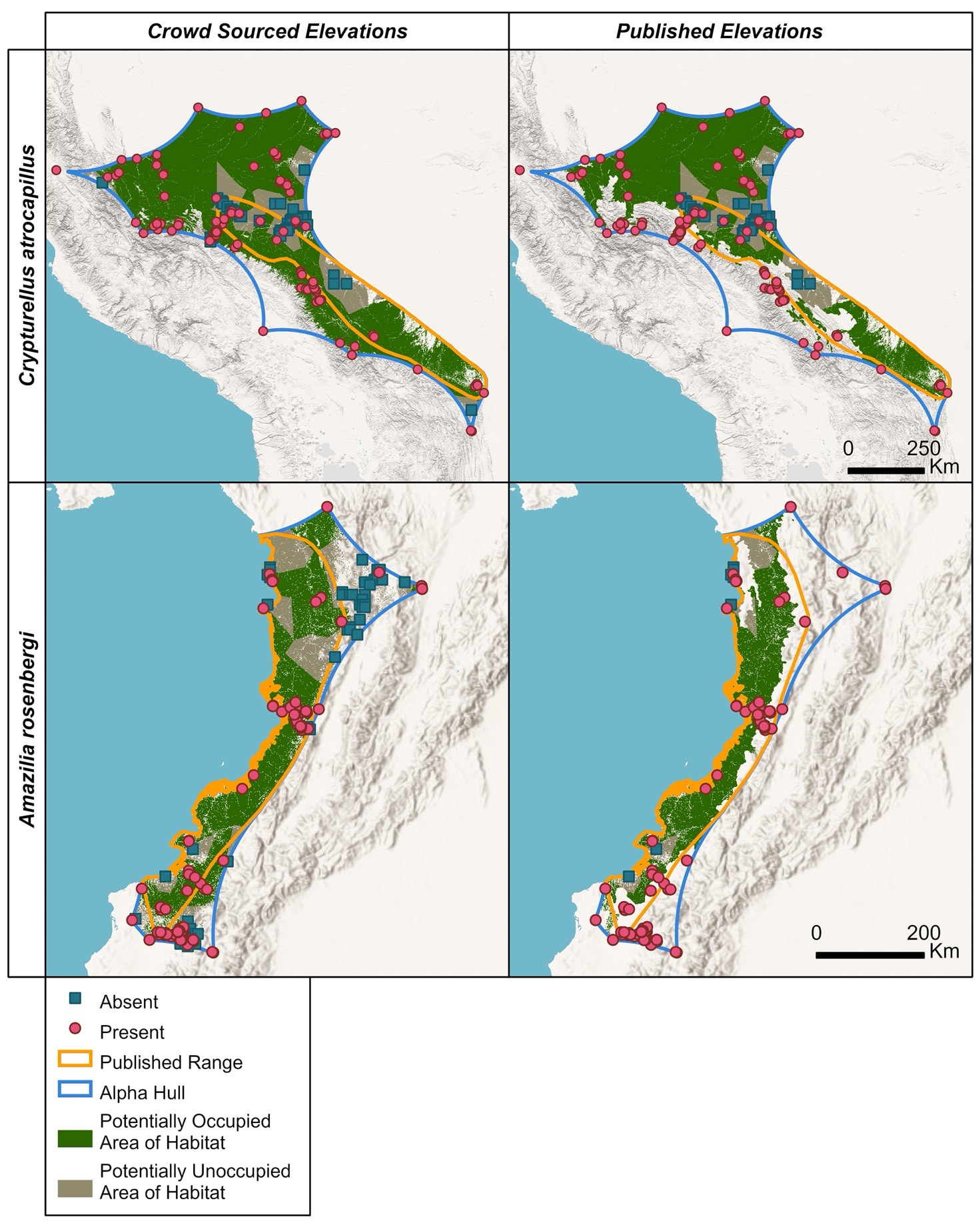
*

**Fig. S3.** Areas of Habitat for *Crypturellus atrocapillus* and *Amazilia rosenbergi* using the published elevations and those derived from this study

Table S1 (Separate file)

Table detailing all target species identified, reason for exclusion from the analysis (if any) and all relevant metrics.

Table S2 (Separate file)

Table of elevational estimates derived from BirdLife, Stotz et al. (1996), or from crowd-source data overlaid on either 90m or 1km DEM.

Table S3 (Separate file)

Comparison of using different estimates of elevational range in calculating Area of Habitat for the eight species with the largest discrepancies.
