# Supplementary material for: Batch-produced, GIS-informed range maps for birds based on provenanced, crowd-sourced data inform conservation assessments
Source: PLoS One. 2021 Nov 24;16(11):e0259299. doi: 10.1371/journal.pone.0259299 (PMC8612558; doi:10.1371/journal.pone.0259299)
Supplement: S1 Code — (PDF) [file pone.0259299.s002.pdf]

Mapping Species Ranges from eBird data

Ryan Huang
Feb 22 2021

Last updated: 2021-03-16 11:10:58

Introduction

What follows is a detailed guideline for batch producing ranges maps for birds using alpha hulls and calculating Area of Habitat. We recommend reading the paper first to gain an understanding of the motivation, advantages, and limitations. We assume that users of this code not only have a basic understanding of R, but also in using eBird's 'auk' package. Ideally, users will be able to adapt the provided code to their use case.

This example will produce a range map for the moss-backed tanager, Bangsia edwardsi, a forest bird native to Ecuador and Colombia. As such, we use the Hansen forest cover data and determine the threshold of forest cover based on the distribution of presence points. However, you may subsitute it for any landcover or other environmental layer you wish to refine by.

Packages

Here is a list of the necessary packages:

```
suppressPackageStartupMessages({
  library(auk)
  library(rgdal)
  library(rgeos)
  library(raster)
  library(sf)
  library(dplyr)
  library(spdplyr)
  library(lubridate)
  library(alphahull)
  library(hull2spatial)
  library(SpatialPosition)
  library(tmaptools)
  library(gstat)
  library(leaflet)
})
```

Data

You will need to provide several external datasets to run this code. The mandatory datasets are a DEM and a raster of habitat which I call `DEM` and `forest` respectively. In Huang et al., we also include a shapefile of published ranges (`publishedRange`) from BirdLife as well as a table of species' elevation ranges (`publishedElev`) also provided by BirdLife.

Projections

You need two coordinate systems, a geographic and a projected coordinate system. The geographic coordinate system is to allow items to be plot via Leaflet. The projected coordinate system is needed to create and buffer the alpha hulls in a later step. Here, we used WGS84 and South America Alber's Equal Area

```
WGS84.proj <- CRS("+proj=longlat +ellps=WGS84 +datum=WGS84 +no_defs")
SAmer.proj <- CRS("+proj=aea +lat_1=-5 +lat_2=-42 +lat_0=-32 +lon_0=-60 +x_
```

Auk

Please refer to the Cornell Lab of Ornithology documentaion for instructions on using the 'auk' package (Strimas-Mackey et al. 2018; https://cornelllabofornithology.github.io/auk/). The code shown here follows the guidelines; `ebd` represents the eBird Basic Dataset and `sed` is the eBird Sampling Event Data.

Checklists

After defining the reference for the eBird data, you must define filters for the checklists that will define your absences. Here I filter eBird checklists to only complete checklists within the extent of the forest data layer. We also remove “Historical” and “Incidental” checklists and “Traveling” checklists greater than 3 hours or 7km due to low confidence in the exact location of the record. The “Traveling” distance limit of 7km also informed the size of the non-detection squares as it is the diagonal of a 5km x 5km square. We will convert each of the checklists into a point at a later step.

```
extent <- extent(forest)
bbox <- c(extent@xmin, extent@ymin, extent@xmax, extent@ymax)
protocols <- valid_protocols[-c(21,23)]

checklists <- sed %>%
  auk_bbox(bbox) %>%
  auk_protocol(protocols) %>%
  auk_complete() %>%
  auk_filter("checklists.txt", overwrite = TRUE) %>%
  read_sampling()

checklists <- subset(checklists, !(protocol_type == "Traveling" & (duration
coordinates(checklists) <- c("longitude", "latitude")
proj4string(checklists) <- WGS84.proj
```

Mapping a Species

All previous steps described only need to be run once per session. From this point on, you need to iterate through all of these steps for each species you intend to map. A simple for loop will work that refers to a list of target species. For our example, we will work with a single species and set `sci.name="Bangsia edwardsi"`

Species Presences

To collect presences, we again use the eBird 'auk'package with the same set of filters as previously. Once we have a data frame of presences, we again convert them to points. A benefit of these methods is that one can interrogate any presence point used to build these maps, either while it is in the data frame, or after it's been converted into spatial points. We have made several decisions in determining which points we are confident in using to build these maps, but anyone who may wish to include or exclude certain presences may simply add or remove these points at this point and execute the code.

```
#This will filter the ebd for the target species. You can also skip this st
species <- ebd %>%
  auk_species(sci.name) %>%
  auk_complete() %>%
  auk_protocol(protocols) %>%
  auk_filter(paste0("Bangsia ewardsi.txt"), overwrite = TRUE) %>%
  read_ebd()

species <- subset(species, !(protocol_type == "Traveling" & (duration_minut
species <- species[,colSums(is.na(species))==0] #Removing excess columns to

#These steps insert columns that will be referencd by the interactive map
species <- species %>%
  mutate(year = year(observation_date)) %>%
  mutate(url = paste0("https://ebird.org/checklist/", checklist_id))

coordinates(species) <- c("longitude", "latitude")
proj4string(species) <- WGS84.proj
```

Alpha Hulls

Once we have the presence points, we create an alpha hull to delineate the extent of interest. Alpha hulls are a generalization of convex hulls (i.e. all convex hulls are alpha hulls but not all alpha hulls are convex hulls) that use a parameter alpha to pinch in empty space without removing points. (The units of alpha are the units of the coordinate system you use, which is why we must project the points). In regions like the Andes where presences may follow a concave shape, the bounding polygon would not include the same amount of empty space as a minimum convex polygon. We have previously loaded the 'alphahull' package written by Pateiro-Lopez (2019) and the 'hull2spatial' package by Babich Morrow (2021).

We also want to ensure our alpha hull includes the published range. However, since alpha hulls do not take polygons as inputs, we need to convert the polygon to points and append them to our eBird presences.

```
presences <- spTransform(species, SAmer.proj)
hull.coords <- presences@coords #We only need to input the coordinates of e

#We recommend setting the alpha-value to the median of the interpresence di
pair.dist<-pointDistance(hull.coords, lonlat = FALSE, allpairs = TRUE)
diag(pair.dist)<-NA
alpha = median(pair.dist, na.rm=T)

poly.p <- spTransform(publishedRange, SAmer.proj)
#Sometimes the polygon sides are long without vertices, so we segment every
poly.p <- as_Spatial(st_segmentize(st_as_sf(poly.p), units::set_units((alph

for (k in 1:length(poly.p@polygons[[1]]@Polygons)){ #This loop is needed if
  poly.coords <- poly.p@polygons[[1]]@Polygons[[k]]@coords
  colnames(poly.coords) <- colnames(hull.coords)
  hull.coords <- rbind(hull.coords, poly.coords)
}
hull.coords <- hull.coords[!duplicated(hull.coords),]

alphaHull<-ahull(x = hull.coords[,1], y = hull.coords[,2], alpha = alpha)
plot(alphaHull) #You can see the raw ahull object bounding the species pres
```

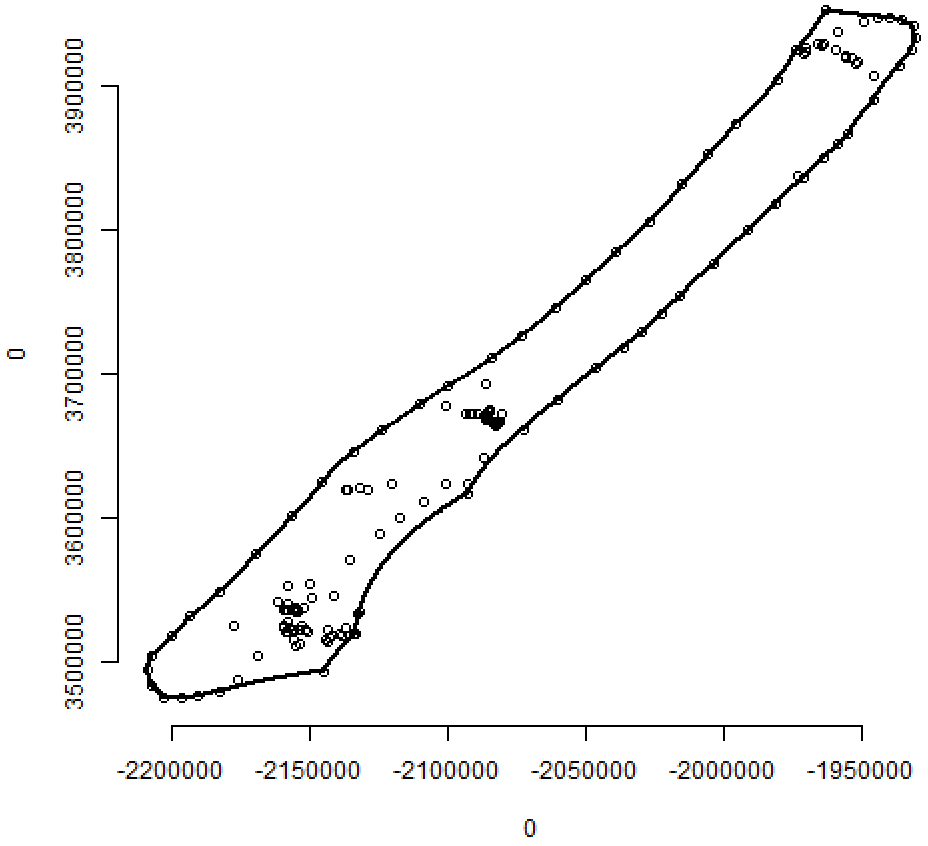


```
alphaHullLines <- ahull2lines(alphaHull)
alphaHullPoly <- spLines2poly(alphaHullLines)
proj4string(alphaHullPoly) <- SAmer.proj
hull <- gBuffer(alphaHullPoly, width = 1000) #Set your buffer width in map
hull <- spTransform(hull, WGS84.proj) #We need to convert back to decimal d
```

Refined Habitat

Recent work has been refining published ranges and Extent of Occurrence in Area of Habitat (AOH) (Ocampo-Pe昼氿uela et al. 2016). Area of Habitat often reflects a more realistic depiction of how much fragmentation and total habitat is available to a species as opposed to a homegeneous polygon. Refining ranges by elevation and landcover are straightforward approaches to do so.

Here, we refine our previously generated alpha hulls using our rasters for DEM and forest (Hansen, 2013). To set thresholds for each, we utilize the distribution of values at our presences, using the middle 98% of elevations and the upper 75% of forest cover percents. Similar to which presence points you wish to use, you may set different thresholds depending on your confidence in the locations of the crowd-sourced data.

```
#Forest Cover
species@data$forest_cover <- raster::extract(forest, species)
spForest <- species@data %>% filter(year>1999) %>% select(forest_cover) #we
spForest <- spForest[spForest>0]
forestThreshold <- unname(quantile(spForest, probs = 0.25))
forest.rcl <- matrix(
  c(0, forestThreshold, forestThreshold, 100,NA, 1),
  nrow = 2, ncol = 3)
forestMask <- raster::mask(forest, hull)

forestMask <- crop(x = forestMask, y = extent(hull))
forestRange <- reclassify(forestMask, forest.rcl, right = FALSE)

#Elevation
species@data$elevation <- raster::extract(DEM, species)
spDEM <- species@data$elevation
DEMLimits <- unname(quantile(spDEM, probs = c(.01,.99)))

#To produce a more conservative estimate, we use the widest range of elevat
#of the distribution of crowd sourced points and the published elevational
if(!is.na(publishedElevMin)){
  DEMmin <- min(c(DEMLimits[1], publishedElevMin))
}else{
  DEMmin <- DEMLimits[1]
}
if(!is.na(publishedElevMax)){
  DEMmax <- max(c(DEMLimits[2], publishedElevMax))
}else{
  DEMmax <- DEMLimits[2]
}

DEM.rcl <- matrix(
  c(DEM@data@min, DEMmin, DEMmax, DEMmin, DEMmax, DEM@data@max, NA, 1, NA),
  nrow = 3, ncol = 3)
DEMmask <- mask(DEM, hull)
DEMmask <- crop(x = DEMmask, y = extent(hull))
DEMRange <- reclassify(DEMmask, DEM.rcl, right = FALSE)

#The following ensures your rasters line up perfectly
if(extent(DEMRange) != extent(forestRange)){
  extent(DEMRange) <- extent(forestRange)
  DEMRange <- resample(DEMRange, forestRange)
}

habitatRefined <- mask(forestRange, DEMRange)
```

Presences vs Absences

Now that we have a refined Area of Habitat, we are interested in leveraging the vast amount of checklists in the eBird dataset. The often-expressed idea that “the absence of evidence is not evidence of absence” has obvious limits. If an area has been extensively surveyed for years without the species having been recorded, it may be indicative of a true absence.

Using eBird's “complete checklists”, we have some sense of how thoroughly an area has been surveyed. We set the threshold for an absence to be 25 complete checklists without a single presence within a 5 km x 5 km grid cell, but this may be set to whatever threshold you believe is appropriate.

This purpose of this section of code is to create a layer of points coded as either a presence, or the center point of an “absence.” Now we will use the `checklists` points we used earlier. The reason we did not generate the list of checklists here is to allow batch processing of many species using a loop without having to generate the checklists every time.

```
#We first select only the checklists within our alpha hull and within appro
sppChecklists <- checklists[hull,]
sppChecklists@data$habitat <- raster::extract(habitatRefined, sppChecklists
sppChecklists <- sppChecklists %>%
  filter(sppChecklists@data$habitat>0)

#We create a blank raster for our extent of interest. This raster will beco
#checklists in our grid cells. You can also think of this as a map of surve
effort <- raster(extent(habitatRefined))
res(effort) = res(habitatRefined)*5 #the habitat raster is 1km and we want
crs(effort) <- crs(habitatRefined)

#Here we tabulate the number of checklists in each grid cells and assign th
effortTable <- table(cellFromXY(effort, sppChecklists))
effort[as.numeric(names(effortTable))] <- effortTable
effort[effort < 25] <- NA #We set our threshold for absences to be 25 check

presenceAbsenceRaster <- effort
ii <- extract(presenceAbsenceRaster, species, cellnumbers=TRUE)[,"cells"]
presenceAbsenceRaster[ii] <- NA #This eliminates any cell where a presence
absenceRaster <- presenceAbsenceRaster #we save the absence raster for disp

#We now convert cells of absences to 0's rather than number of checklists w
absenceCells <- extract(presenceAbsenceRaster, sppChecklists, cellnumbers=T
ii <- subset(absenceCells, !is.na(absenceCells[,"layer"]))[,"cells"]
presenceAbsenceRaster[ii] <- 0

#Below we convert the absence raster cells into points at the center and co
#we are left with points with values of 1 and 0 for presences and absences
absencesPts <- data.frame(coordinates(presenceAbsenceRaster), Presence=pres
absencesPts <- absencesPts[!is.na(absencesPts$Presence), ]
if(length(absencesPts$Presence)>0){
  coordinates(absencesPts) <- c("x", "y")
  proj4string(absencesPts) <- proj4string(habitatRefined)
  presenceAbsencePts <- matrix(
    c(absencesPts@coords[,1], species@coords[,1],
      absencesPts@coords[,2], species@coords[,2],
      rep(0, length(absencesPts@coords[,1])),rep(1, length(species@coords[,
    ncol = 3)
  presenceAbsencePts<- presenceAbsencePts[!duplicated(presenceAbsencePts[,1
}else {
  presenceAbsencePts <- matrix(
    c(species@coords[,1],
      species@coords[,2],
      rep(1, length(species@coords[,1]))),
    ncol = 3)
```

```
}
colnames(presenceAbsencePts) <- c("Long", "Lat","Presence")
presenceAbsencePts <- data.frame(presenceAbsencePts)
coordinates(presenceAbsencePts) <- c("Long", "Lat")
proj4string(presenceAbsencePts) <- WGS84.proj
```

Nearest Neighbor Interpolation

Now that we have spatial points with values to represent presences or absences, we want to identify every pixel in our extent of interest that is closer to a presence or an absence. We define Area of Habitat closer to a presence as Potentially Occupied Area of Habitat (POAOH). We can acheive this Using a simple, unweighted nearest neighbor interpolation.

```
nn.rcl <- matrix(
  c(0, 0.5, 0.5, 1,NA, 1),
  nrow = 2, ncol = 3)

blankRaster <- raster(extent(habitatRefined))
res(blankRaster) = res(habitatRefined)
crs(blankRaster) <- WGS84.proj
gs <- gstat(formula = presenceAbsencePts$Presence ~ 1, locations = presence
nearestNeighbor <- interpolate(blankRaster, gs)
```

```
## [inverse distance weighted interpolation]
```

```
nearestNeighbor <- reclassify(nearestNeighbor, nn.rcl, right = FALSE)

POAOH<- mask(habitatRefined, nearestNeighbor)
```

Mapping Our Results

We now have all the pieces to visualize the final maps. We have the presences, the “absences”, the alpha hull, the Area of Habitat, and the Potentially Occupied Area of Habitat. We will now put these together in a interactive Leaflet map. This is not meant to be a tutorial in using Leaflet, so if you wish to learn more, you can find the documentation here: https://rstudio.github.io/leaflet/

The major benefit of creating an interactive map is to allow the interrogation of all the underlying data. You can zoom in and out, click on absent grid cells to see the number of checklists without a presence, or click on any presence point to view the date of observation, the elevation, forest cover %, or even link to the eBird checklist for further investigation. The

resulting map not only makes the process more transparent by seeing what data has been included, but it also allows anyone to make decisions as to which data points to include or exclude.

```
#First we will set the colors and symbology
#colors
presence.col <- "#a9eb75"
AOH.col <- "#808080"
POAOH.col <- "#0d4201"
published.col <- "#d6872d"
hull.col <- "#6CD4FF"

# Legend
legend.values = c("Presence Points", "Area of Habitat", "Possible Area of H
legend.col = c(presence.col, AOH.col, POAOH.col, published.col , hull.col)
```

```
#Now we will create the Leaflet map
suppressWarnings(range_map <- leaflet() %>%
                   addProviderTiles(providers$Esri.WorldTopoMap) %>%
                   addRasterImage(habitatRefined, colors = AOH.col, opacity
                   addRasterImage(POAOH, colors = POAOH.col, opacity = 1) %
                   addPolygons(data = hull, stroke = TRUE, color = hull.col
                   addPolygons(data = publishedRange, color = published.col
                   #This next line controls the ability to turn layers on a
                   addLayersControl(overlayGroups = c("Published Range","No
                                    options = layersControlOptions(collapse
                   #This mini map provides an inset map for context
                   addMiniMap(
                     tiles = providers$Esri.WorldTopoMap,
                     width = 150,
                     height = 150) %>%
                   addLegend("topright",
                             colors = legend.col,
                             labels = legend.values,
                             opacity = 1,
                             title = paste(sci.name, "<br> Alpha =",floor(a
                   addScaleBar(options = scaleBarOptions(metric = TRUE, imp

#We will also convert the absence raster into polygons to interact with
absencePolygon <- rasterToPolygons(absenceRaster)
absenceLabels <- absencePolygon@data$layer

absence.col <- colorNumeric(
  palette = "YlOrRd",
  domain = absencePolygon$layer)

range_map <- range_map %>%
  addPolygons(data = absencePolygon,
              fillColor = ~absence.col(layer),
              color = ~absence.col(layer),
              opacity = 1,
              weight = 1,
              fillOpacity = 0.75,
              popup = ~paste("<b>Absences: </b>",layer,"<br>"),
              group = "Non-Detection") %>%
  addLegend("bottomleft", pal = absence.col, values = absencePolygon$layer,
            title = "Number of Absences",
            opacity = 1)

#Lastly we will add the presences
range_map <- range_map %>%
  addCircleMarkers(data = species,
```

```
                    radius = 4,
                    fillColor = presence.col,
                    fillOpacity = 1,
                    stroke = TRUE,
                    opacity = 1,
                    weight = 1,
                    #This is where Leaflet reads the attributes for each poi
                    popup = ~paste("<b>Protocol: </b>", protocol_type, "<br>
                                   "<b>Date: </b>", observation_date, "<br>"
                                   "<b>Elevation (m): </b>", elevation, "<br
                                   "<b>Forest Cover (%): </b>", forest_cover
                                   "<b>Locality: </b>", locality, "<br>",
                                   "<b>Checklist: </b>","<a href='",url,"' t
                    color = "#4d7e29",
                    group = "eBird Presences")
```

```
range_map
```

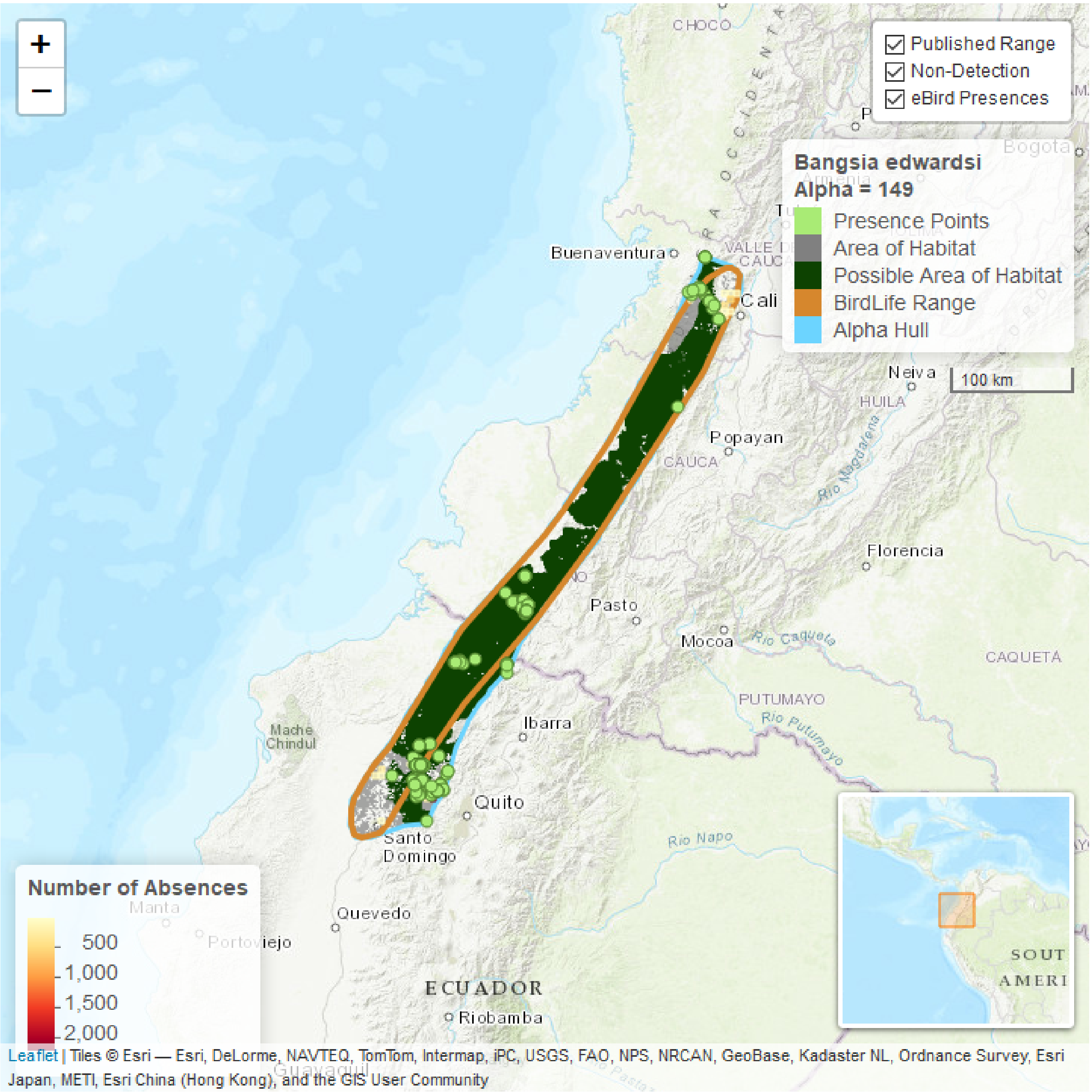

References

M. Strimas-Mackey, E. Miller, and W. Hochachka (2018). auk: eBird Data Extraction and Processing with AWK. R package version 0.3.0. https://cornelllabofornithology.github.io/auk/

B. Pateiro-Lopez and A. Rodriguez-Casal (2019). alphahull: Generalization of the Convex Hull of a Sample of Points in the Plane. R package version 2.2. https://CRAN.R-project.org/package=alphahull

C. Babich Morrow (2021). hull2spatial: Convert Alpha Hulls to Spatial* Objects. R package version 0.1.0.

N. Ocampo-Pe昼牠uela, C. N. Jenkins, V. Vijay, B. V. Li, and S. L. Pimm (2016). Incorporating explicit geospatial data shows more species at risk of extinction than the current Red List. Science Advances 2, e1601367 .
